# A Review of Current and Future Pharmacologic Treatments for Narcolepsy

**DOI:** 10.1192/j.eurpsy.2024.1618

**Published:** 2024-08-27

**Authors:** P. Chue, J. Chue, M. Tate, A. Andreiev, A. Abba-Aji

**Affiliations:** ^1^Psychiatry, University of Alberta; ^2^Clinical Trials and Research Program, Edmonton, Canada

## Abstract

**Introduction:**

Narcolepsy is a rare but disabling neurological disorder involving disruption of the sleep-wake cycle that is often under- or misdiagnosed (Barateau L, *et al*. J Sleep Res. 2022;31(4):e13631). It is characterized by a classical tetrad of excessive daytime sleepiness (EDS), cataplexy, hypnagogic hallucinations, and sleep paralysis. Narcolepsy is divided into 3 types: Narcolepsy Type 1 (NT1); Narcolepsy Type 2 (NT2); and Secondary Narcolepsy. The pathophysiology remains unclear but is primarily associated with loss of hypocretin (orexin) neurons involving autoimmune and genetic risk factors, particularly for NT1.

**Objectives:**

To review the currently available therapies for the treatment of narcolepsy.

**Methods:**

The extant literature was reviewed and discussed in the context of clinical relevance.

**Results:**

Treatment historically has included medications developed for the treatment of other conditions such as psychostimulants (methylphenidate, modafinil/armodafinil, pemoline) and antidepressants (SSRIs,TCAs). These agents are also associated with limiting side effects in practice. In more recent years a variety of specific treatments have been approved that act on diverse pathways. Pitolisant, a histamine H3 receptor inverse agonist, is approved for the treatment of EDS or cataplexy in adult patients with narcolepsy (and children> 6 years in European Union) (Keam SJ.Paediatr Drugs. 2023;25(4):483-488). Solriamfetol, a dopamine and norepinephrine reuptake inhibitor (DNRI) is indicated to improve wakefulness in adult patients with EDS associated with narcolepsy or obstructive sleep apnea (OSA) (Winter Y, *et al.* Sleep Med. 2023;103:138-143). Sodium oxybate (SXB), a GABA_B_ receptor agonist, is approved for the treatment of cataplexy associated with narcolepsy and (EDS) in patients 7 years or older (Bogan RK, *et al.* CNS Drugs. 2023;37(4):323-335). Current research focuses on on-peptide hypocretin receptor-2 agonists (Saitoh T, Sakurai T. Peptides. 2023;167:171051).

**Conclusions:**

Despite limited understanding of the pathophysiology of narcolepsy there have been substantial advances in the pharmacotherapy, including medications now approved for children. Early diagnosis and treatment are associated with better outcomes. In view of the chronic and disabling morbidity associated with narcolepsy further research and better access to appopriate medications is necessary.

**Disclosure of Interest:**

None Declared

